# Stability of immediately loaded 3 mm long miniscrew implants: a feasibility study

**DOI:** 10.1590/2177-6709.26.1.e2119155.oar

**Published:** 2021-03-22

**Authors:** Danielle GESHAY, Phillip CAMPBELL, Larry TADLOCK, Emet SCHNEIDERMAN, Hee-Moon KYUNG, Peter BUSCHANG

**Affiliations:** 1 Private practice (Dallas/TX, USA).; 2 Texas A&M University, College of Dentistry, Department of Orthodontics (Dallas/TX, USA).; 3 Texas A&M University, College of Dentistry, Department of Biomedical Sciences (Dallas/TX, USA).; 4 Kyungpook National University, Department of Orthodontics (Daegu, Korea).

**Keywords:** Miniscrew implants, Stability, Pain/discomfort, Experience

## Abstract

**Introduction:**

Shorter miniscrew implants (MSIs) are needed to make orthodontics more effective and efficient.

**Objective:**

To evaluate the stability, insertion torque, removal torque and pain associated with 3 mm long MSIs placed in humans by a novice clinician.

**Methods:**

82 MSIs were placed in the buccal maxillae of 26 adults. Pairs of adjacent implants were immediately loaded with 100g. Subjects were recalled after 1, 3, 5, and 8 weeks to verify stability and complete questionnaires pertaining to MSI-related pain and discomfort.

**Results:**

The overall failure rate was 32.9%. The anterior and posterior MSIs failed 35.7% and 30.0% of the time, respectively. Excluding the 10 MSIs (12.2%) that were traumatically dislodged, the failure rates in the anterior and posterior sites were 30.1% and 15.2%, respectively; the overall primary failure rate was 23.6%. Failures were significantly (*p*= 0.010) greater (46.3% vs 19.5%) among the first 41 MSIs than the last 41 MSIs that were placed. Excluding the traumatically lost MSIs, the failures occurred on or before day 42. Subjects experienced very low pain (2.2% of maximum) and discomfort (5.5% of maximum) during the first week only.

**Conclusions:**

Shorter 3 mm MSIs placed by a novice operator are highly likely to fail. However, failure rates can be substantially decreased over time with the placement of more MSIs. Pain and discomfort experienced after placing 3 mm MSIs is minimal and temporary.

## RESUMO

**Introdução:**

Mini-implantes (MIs) mais curtos são necessários para uma Ortodontia mais eficiente e efetiva.

**Objetivo:**

Avaliar a estabilidade, torque de inserção e de remoção e dor associada a MIs de 3mm instalados em humanos por um ortodontista principiante.

**Métodos:**

82 MIs foram instalados na região vestibular da arcada superior de 26 adultos. Pares de mini-implantes adjacente receberam carga imediata de 100g. Após 1, 3, 5 e 8 semanas, os pacientes foram reavaliados para verificar a estabilidade e preencher um questionário sobre a dor e o desconforto relacionados aos MIs.

**Resultados:**

A taxa geral de falhas foi de 32,9%, sendo de 35,7% para os MIs anteriores e 30% para os MIs posteriores. Excluindo os 10 MIs que foram perdidos por trauma (12,2%), a taxa de falha nas regiões anterior e posterior foram de 30,1% e 15,2%, respectivamente e ocorreram no 42^0^ dia ou antes. A taxa geral de falha primária foi de 23,6%. A taxa de falha foi significativamente maior (p=0,010) nos primeiros 41 MIs do que nos 41 últimos (46,3% vs. 19,5%). As experiências relacionadas à dor foram baixas (2,2% máximo), assim como ao desconforto (5,5% máximo) durante a primeira semana.

**Conclusão:**

MIs de 3mm instalados por um novato são mais propensos a falhas. Porém, as taxas de falha podem diminuir substancialmente com a instalação de mais MIs com o decorrer do tempo. A dor e o desconforto após a instalação desses dispositivos são mínimos e temporários.

## INTRODUCTION

Miniscrew implants (MSIs) have become popular due to their easy placement and removal, effectiveness as anchorage devices, the multiplicity of intraoral placement options, their affordability, minimal invasiveness, and patient acceptance.^1,2^ Systematic reviews have reported failure rates ranging between 13.5 and 20% when mobile and displaced MSIs were included.^3^ ^,^ ^4^ Approximately 85% of practicing orthodontists reported MSI failures of 25% or less.^5^ Although root injuries caused during MSI placement usually heal unremarkably, they have been shown to cause localized bone loss, ankylosis, and pulpal damage, leading to devitalization of the tooth.^6^ ^-^ ^8^

The risk of root contact during placement should be less for shorter 3 mm long MSIs than for traditional 6 to 8 mm long MSIs. Reports have shown that the soft tissue adjacent to the mucogingival junction is approximately 1-1.5 mm thick,^9^ ^,^ ^10^ and that interradicular cortical bone is 0.8-3.1 mm thick in the mandible and 0.8-1.5 mm thick in the maxilla.^11^ ^,^ ^12^ On that basis, no more than 1.5 mm of the screws’ shanks would penetrate into medullary bone. That being the case, the risk of root contact in the buccal posterior region with 3 mm long MSIs should be minimal. For the same reasons, shorter screws should provide orthodontists with more MSI placement options, reduce the need for root separation, and could provide skeletal anchorage for dentofacial orthopedics in younger patients.

Experimental studies have shown that 3 mm long MSIs are stable in animal models. Excluding problematic MSIs (i.e. those whose tips broke off during insertion and those traumatically dislodged by animals chewing on their cages), an overall failure rate of 9.4% was reported for 3 mm screws placed in dog jaws and loaded with orthopedic level forces.^13^ Failure rates under 10% have been reported in studies that placed 3 mm MSIs in the cranium of rabbits and loaded them with various orthodontic level forces.^14^ ^-^ ^17^

The purpose of this study was to assess the stability of immediately loaded 3 mm miniscrew implants placed in human subjects by a novice operator. No studies to date have examined the feasibility of using 3 mm long, 1.7 mm wide, MSIs in humans. Moreover, little is known about the number of MSIs that novice operators have to place in order to attain acceptable failure rates.

## MATERIAL AND METHODS

### SUBJECTS

**Figure 1:**
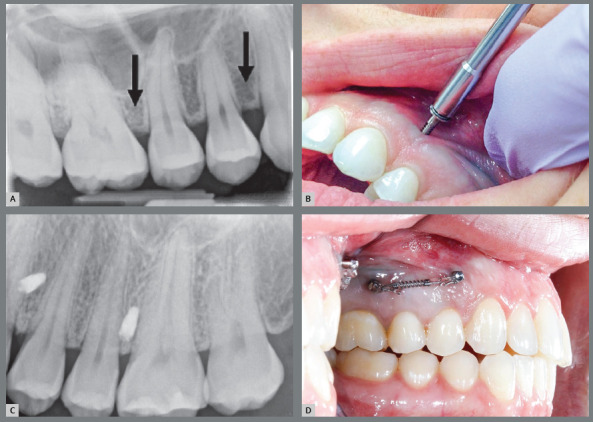
A) Periapical radiograph taken to visualize bone between the teeth (planned MSI sites indicated by black arrows), B) insertion of MSI, C) periapical radiograph taken after MSI placement, D) pair of MSIs after immediate loading with a force of 100 g.

After receiving approval by the Institutional Review Board, adults were recruited from the students and staff at Texas A&M University College of Dentistry. Exclusion criteria included: 1) pregnant females, 2) smokers, 3) subjects taking medications that could affect bone metabolism, 4) inadequate space between tooth roots and 5) buccal frenum in the placement site. Periapical radiographs were taken to ensure sufficient space between the MSIs and tooth roots (Fig 1).

A power analysis indicated that 80 screws were needed to establish a 15% difference in stability, assuming a power of 95% and an alpha of 0.05. A total of 82 MSIs were placed in 26 subjects (10 males, 16 females) with a mean age of 27.4 years. Each subject received $25 to compensate for their time and efforts. Seventeen subjects had MSIs placed on both sides (17*x*4 = 68), 6 had only one side that qualified (6*x*2 = 12), and 2 had only one (unloaded) MSI placed. The anterior MSI was placed between the maxillary canines and first premolars; the posterior MSI was placed between the second premolars and first molars. The protocol was approved by the Texas A&M University IRB. The purpose of the study and potential risks were explained, and an informed consent was obtained from each subject.

### PLACEMENT PROTOCOL

All procedures were performed by one orthodontic resident using a standardized placement protocol. Subjects brushed their teeth and rinsed with Peridex chlorhexidine (3M ESPE, Irvine, CA) for 45 seconds. Topical anesthesia (20% lidocaine, 4% tetracaine, 2% phenylephrine) was applied at each MSI site for two minutes. Three subjects who requested additional anesthesia received anesthetic infiltration with 1/8 carpule of 2% lidocaine with 1:100,000 epinephrine. Gingival thickness was measured three times at the insertion site using a sharp explorer with an endodontic rubber stop. 

Each 3 mm long, 1.7 mm wide MSI (Dentos, Seoul, Korea) was inserted perpendicularly into bone with a manual driver until the screw threads were no longer visible. Using a digital torque screwdriver (Imada, Northbrook, IL), each MSI was then rotated a quarter turn to measure insertion torque. 

Periapical radiographs were taken after placement to ensure that there was space between the MSIs and the adjacent bone. Pairs of adjacent implants were immediately loaded with one nickel-titanium closed-coil spring (Ormco, Orange, CA) delivering a force of 100g. Triad gel (Dentsply, York, PA) was applied to prevent wire abrasion of the cheeks or gingiva. Following placement, subjects rinsed with Peridex for 45 seconds, and were instructed to rinse each night for one week. Intraoral photographs were taken and each subject was given written oral hygiene and miniscrew care instructions. Orthodontic wax was given to each subject to prevent cheek irritation. The distance between each pair of MSIs was measured with a caliper on the day of placement, as well as 1, 3, 5 and 8 weeks after placement. The distance was measured three times and averaged at each occasion. 

MSIs were considered as failures if they exhibited any degree of mobility upon examination. If a screw failed, it was replaced if there was sufficient space intraorally to relocate the MSI apically. Screws were replaced whenever possible to maintain the 100g force on the other MSI that did not fail. Importantly, MSIs that were replaced were counted as failures. If both screws on one side failed, they were removed and not replaced. Failures were classified as either primary or incidental. Incidental failures occurred when the MSIs were traumatically dislodged by the subjects. After eight weeks, removal torque was recorded based on the first counterclockwise turn of each MSI using the digital torque screwdriver. 

### FOLLOW-UP

Subjects were recalled after 1, 3, 5, and 8 weeks. The eight week duration was chosen because the majority of MSI failures occur within one month after placement.^18^ ^-^ ^20^ At each appointment, miniscrew stability was verified and the distance between implants was measured. Subjects also completed a questionnaire asking them to rate the worst pain that they ever experienced and the pain they were currently feeling, using a 10-cm Visual Analog Scale (VAS) anchored with “No pain” and “Worst pain ever”. Another question, anchored with “No discomfort” and “Worst discomfort ever”, asked how much discomfort the subjects were currently experiencing. The next two questions asked whether they took medications to relieve pain or discomfort associated with, and not associated with, the MSIs. The final question asked if the miniscrew implants caused any type of injury. 

### STATISTICAL ANALYSIS

SPSS Statistics version 22 (SPSS Inc, Chicago IL) was used for data analysis. Insertion torque, removal torque, and MSI distance data were analyzed using paired samples *t*-tests. Failures were evaluated using Chi-Square tests. Timing of failures was evaluated using non-parametric Mann-Whitney tests and differences between time points were compared using 2-tailed Wilcoxon Signed Rank tests. The survey responses were evaluated using Friedman tests. Statistical significance for all data was set at *p*< 0.05.

## RESULTS

### FAILURES

An implant was considered a failure if it exhibited any mobility upon examination (Table 1). The overall failure rate was 32.9% (27/82). Neither of the two unloaded MSIs failed. The failure rates of the anterior and posterior screws were 35.7% (15/42) and 30.0% (12/40), respectively. Ten of the 27 failures were incidental failures, where the MSIs were unintentionally but traumatically displaced by the subjects. The remaining failures were primary failures. The overall primary failure rate was 23.6% (17/72). The primary failure rates for the anterior and posterior screws were 30.1% (12/39) and 15.2% (5/33), respectively. 

**Table 1:** Miniscrew failure rate.

	All failures (primary and incidental failures)	Primary failures (excluding incidental failures)
All MSIs	27/82 (32.9%)	17/72 (23.6%)
Anterior MSIs	15/42 (35.7%)	12/39 (30.1%)
Posterior MSIs	12/40 (30.0%)	5/33 (15.2%)

**Figure 2:**
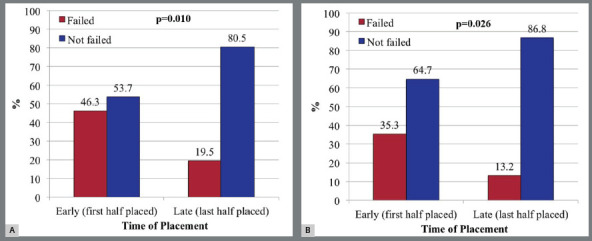
Percentages of MSI failures for those placed early (i.e., the first half of MSIs placed by investigator) and late (i.e., the last half), A) including all (82 total) and B) excluding incidental failures (72 total).

There was no significant difference in failure rate between MSIs placed on the right and left sides (Table 2) or between anterior and posterior screws. There were significantly more failures among the first 41 screws placed by the investigator than among the last 41, when all screws were considered and when incidentally displaced screws were excluded (Fig 2). 

**Table 2:** Factors potentially associated with MSI failure.

Factor		Failed	Not failed	Sig.
Side	Right	14/46	32/46	0.587
		(30.4%)	(69.6%)	
	Left	13/36	23/36	
		(36.1%)	(63.9%)	
AP (all screws - 82 total)	Anterior	15/42	27/42	0.582
		(35.7%)	(64.3%)	
	Posterior	12/40	28/40	
		(30%)	(70%)	
AP (excluding incidental failures - 72 total)	Anterior	12/39	27/39	0.120
		(30.8%)	(69.2%)	
	Posterior	5/33	28/33	
		(15.2%)	(84.8%)	
	Late (last ½ placed)	5/38	33/38	
		(13.2%)	(86.8%)	

**Figure 3:**
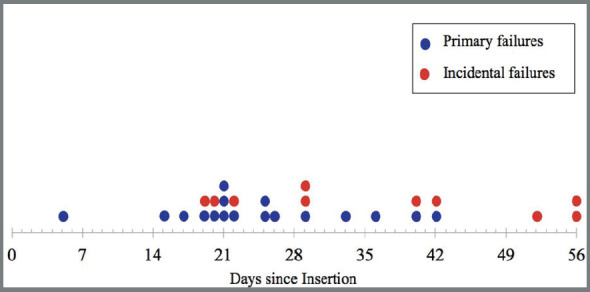
Days from the beginning of the study at which the primary and incidental failures occurred.

All primary failures occurred on or before day 42 (Fig 3); most failed between 15 and 26 days. Incidental failures, which began on day 19, displayed no clear pattern and continued throughout the eight-week study.

### INSERTION AND REMOVAL TORQUE

Insertion torque was 7.8 ± 1.2 Ncm for the anterior screws and 7.4 ± 1.9 Ncm for the posterior screws, with no statistically significant anteroposterior difference (Table 3). There also was no statistically significant difference in removal torque between the anterior (1.7 ? 0.9 Ncm) and posterior (1.7 ? 0.7 Ncm) screws. Insertion torque was significantly (*p*< 0.01) greater than removal torque. While insertion and removal torque were higher for the MSIs that failed than for those that did not fail, none of the differences were statistically significant differences. However, insertion and removal torque were significantly less for the first 41 MSIs than the last 41 MSIs that were placed (Table 4).

**Table 3:** Mean MSI insertion and removal torque at 56 days.

		Insertion torque (Ncm)	Removal torque (Ncm)	Diff.
Anterior MSIs	Mean	7.75	1.71	p < 0.001
	SD	1.24	0.94	
Posterior MSIs	Mean	7.39	1.69	p < 0.001
	SD	1.92	0.70	
Diff.		p = 0.193	p = 0.686	

Bold terms indicate significance (*p* < 0.05).

**Table 4:** Differences in insertion and removal torque between the first and last 41 MSIs placed.

		First 41 MSIs		Last 41 MSIs		
Torque	MSIs	Mean	SD	Mean	SD	prob
Insertion (Ncm)	Anterior	7.29	1.02	8.23	1.12	<0.001
	Posterior	6.78	2.03	7.98	1.65	0.006
Removal (Ncm)	Anterior	1.47	1.11	1.80	0.86	0.32
	Posterior	1.42	0.70	1.96	0.59	0.003

### GINGIVAL THICKNESS & TIPPING

**Figure 4:**
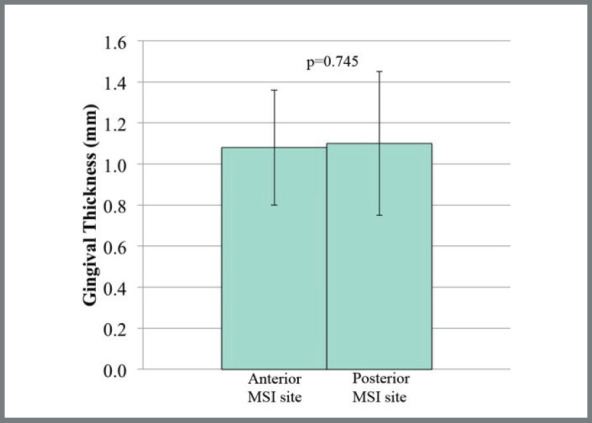
Gingival thickness measurements (mean ± SD) at the anterior and posterior MSI insertion sites.

Mean gingival thickness was 1.1 ? 0.3 mm at the anterior insertion sites, and 1.1 ??#8197;0.1 mm at the posterior insertion sites (Fig. 4), a difference that was not statistically significant (*p*= 0.745). There were no statistically significant differences in gingival thickness between MSIs that failed and did not fail, or between the first 41 MSIs placed and the last MSIs placed.

**Figure 5:**
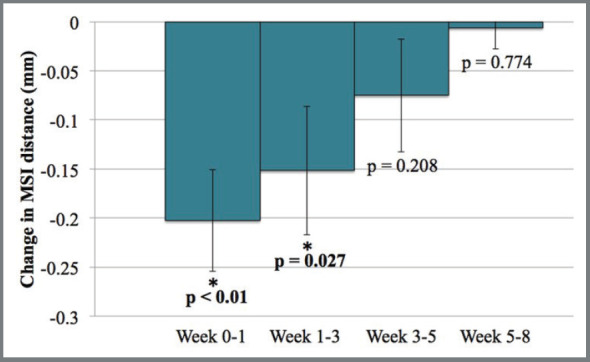
Mean change in distance between adjacent MSIs at each time interval (± standard error of the mean).

The distances between pairs of adjacent MSIs decreased over time (Fig 5). Statistically significant decreases occurred between placement and week 1 (*p*< 0.01), as well as between weeks 1 and 3 (*p*= 0.027). Decreases thereafter were small and not statistically significant. While there were no statistically significant differences in the distances that the screws moved during the first three weeks between the first and last 41 MSIs placed, there were significantly greater movements of the MSIs that failed (Table 5).

**Table 5:** Distances moved over the first 3 weeks of MSIs that failed and did not fail, with estimated for all, including and excluding incidental failures.

		Failed MSIs		Not Failed		
Torque	Weeks	Mean	SD	Mean	SD	prob
Including all screws	0-3	-0.64	0.48	-0.25	0.42	0.003
Excluding incidental failures	0-3	-0.69	0.40	-0.25	0.42	0.006

### QUESTIONNAIRES

Responses to the first question, regarding worst pain ever experienced, ranged from 74.0 to 76.9, with no statistically significant differences between the four time points (Table 6). Significant (*p*< 0.01) decreases in current pain and discomfort were reported between weeks 1 and 3, with no statistically significant changes thereafter. 

**Table 6:** Pain and discomfort [medians (Med) and interquartile ranges] associated with MSIs at follow-up evaluations, measured on a Visual Analog Scale.

	Week 1			Week 3			Week 5			Week 8		
%	25%	Med	75%	25%	Med	75%	25%	Med	75%	25%	Med	75%
Worst pain ever*	57.9	75.3	80.8	59.0	74.6	82.1	53.4	74.0	83.9	58.4	76.9	82.9
Current Pain	0.0	2.2	8.2	0.0	0.3	2.8	0.0	0.0	1.9	0.0	0.0	1.3
Current Discomfort	2.6	5.5	15.9	0.0	1.0	3.9	0.0	0.5	2.9	0.0	0.0	2.2

*Indicates the worst pain ever experienced by the subject prior to study participation.

The percentages of subjects taking medication to relieve MSI-associated pain or discomfort decreased significantly (*p*< 0.01), from 61.6% at week 1 to 4.2% at week 3. Approximately 2-29% of subjects reported taking analgesics for pain unrelated to the MSIs, with no statistically significant differences between time points. 46.2% of respondents indicated that the MSIs caused injuries during the first week, including cheek rubbing and mucosal ulceration (n = 8), gingival sloughing due to topical anesthetic (n = 2), and gingival irritation due to the coil spring (n = 2). One subject reported injury on week 5, due to a small cheek ulceration and another subject, who had a mobile screw, reported painful and swollen gingiva at week 8. 

## DISCUSSION

In the present study, 10 screws were traumatically dislodged by the subjects. These were the “incidental failures” that mostly (70%) occurred when subjects bit into large or tough foods. One anterior screw was traumatically displaced during tooth brushing. An informal survey of four clinical orthodontists, who together have over 42 years of experience using MSIs in their practices, indicated very few, if any, trauma-related failures. Fewer incidental failures might be expected among orthodontic patients because their diets are typically softer.^21^ Also, the profile of orthodontic brackets and wires shields MSIs from food boluses during mastication and displaces the cheeks/lips of the gingiva. Since incidental trauma-related failures are expected to be less likely among orthodontic patients, both the total failures and the primary (i.e. non-traumatic) failures must be considered. Since 3 mm MSIs may pose a greater risk of choking than longer MSIs, it is important that they remain attached to appliances at all times.

The failure rate of 3 mm MSIs placed in the present study by a novice operator was substantially higher than rates reported for longer screws. The overall and primary failure rates were 32.9% and 23.6%, respectively. The most recent comprehensive systematic review of the literature indicates that approximately 13.5% of MSIs failed.^4^ However, the review included studies with mobile screws, screws of various designs (several as long as 17 mm) placed throughout the oral cavity, and screws used for a variety of purposes. 

While there are no comparative human studies using 3 mm MSIs, experimental animal studies have reported good success rates. The overall failure rate of loaded 3 mm MSIs placed in dog jaws was 9.4% after excluding MSIs whose tips broke off while being inserted and MSIs that were traumatically dislodged.^13^ Studies that placed 3 mm MSIs in the cranium of rabbits and loaded them with various expansion forces reported failure rates of less than 10%.^14^ ^-^ ^17^ The marked discrepancy between human and animal failure rates suggests that the stability of 3 mm MSIs depends on factors other than length.

Only two clinical studies evaluated shorter MSIs placed in the posterior buccal maxillary segment of humans. Suzuki et al.^22^ reported a failure rate of 6.6%, with no differences in stability between immediately-loaded 5, 6, and 7 mm MSIs. In contrast, a 24.8% failure rate was reported for 5 mm MSIs that were loaded after 2-3 weeks.^23^ The difference between these two studies further supports the notion that factors other than length determine the stability of shorter MSIs. 

The insertion site could have been one factor that explains the higher than expected failure rates. Although not statistically significant, the primary failure rate for the anterior screws was twice as high (30.1 *vs* 15.2%) as the rate for MSIs placed between the second premolar and first molar. The anterior screws were often placed in non-keratinized movable mucosa, which is a known risk factor for miniscrew failure.^24^

Clinical experience was an important determinant of MSI success. The first 41 screws placed were much more likely to fail than those placed during the second half of the study (46.3%*vs*19.5% failure). The primary failure rates during the last half was only 13.2%, which is similar to or less than failure rates reported for longer screws.^3^ ^-^ ^5^ Experience explains why others have reported lower MSI failure rates,^25^ ^,^ ^26^ and why failure rates are less among professors than postgraduate students (1.9% *vs* 29.2%).^27^ A novice operator’s failure rates with 5 mm MSIs decreased from 25% during the first 18 months to 8.8% during the next 18 months.^28^ The significantly lower insertion and removal torque found in the present study for the first 41 MSIs placed suggests that the bone was damaged to a greater extent and required more healing. This could have been due to greater speed of insertion or less stability (e.g. wobble) during the insertion process. The greater movements observed between MSIs that failed also indicates less primary stability.

Failures of 3 mm MSIs mostly occurred two to four weeks after insertion. This confirms retrospective studies showing most MSI failures occurring during the first month after placement.^18^ ^-^ ^20^ Dog studies reveal that MSI stability decreases during the first three weeks, and then increases.^29^ ^,^ ^30^ Stability decreases because damaged bone must be removed during the initial stages of healing; stability increases after 3-4 weeks as bone deposition and remodeling surpass the resorption of the old bone.

Insertion torque indicated that the primary stability of 3 mm MSIs is similar to the primary stability of longer screws.^31^ ^,^ ^32^ Higher insertion torques have been reported for longer self-drilling MSIs.^24^ Insertion torque in the present study was within 5-10 Ncm recommended for MSIs.^21^ Shorter and longer screws likely exhibit comparable insertion torque because primary stability depends mostly on the cortical thickness and density.^33^

Removal torque of the 3 mm MSIs demonstrated reduced secondary stability. The average removal torque for the 3 mm MSIs was 1.7 Ncm, which is lower than removal torques previously reported for longer screws.^18^ ^,^ ^32^ This was primarily due to the short eight week healing time in the present study. Also, shorter screws have less surface area than longer screws and less bone-to-implant contact for osseointegration. Bone forms along the entire surface of MSIs during the healing phase.^34^ ^,^ ^35^

In the present study, the distances between pairs of implants decreased significantly during the first three weeks. Clinically, the change was minimal, with an average total decrease of only 0.5 mm. Longer 17 mm MSIs have been reported to tip after placement,^36^ ^,^ ^37^ with the amount being related to the amount of force applied.^13^ The majority of MSI tipping probably occurs during the first few weeks, before newly remodeled bone achieves intimate contact with the MSI threads.^30^

Pain and discomfort experienced after MSI insertion was minimal. There was only slight pain and discomfort after the first week, and little or none thereafter. While patients expect MSIs to be moderately or very painful before placement, they report no pain or mild pain after treatment.^38^ Longer (6-12 mm) MSIs produce less than half as much pain as traditional orthodontic appliances.^39^ ^,^ ^40^ Pain could be even further reduced with the shorter 3 mm MSIs because they are less likely to contact the periodontal ligament, assuming that less than 1.5 mm of the screw’s shank is expected to penetrate the medullary bone. Pain for the subjects in the present study was due to soft tissue injuries caused by MSI placement, including cheek rubbing, ulceration, gingival sloughing from topical anesthetic, and gingival irritation from the coil springs. 

## CONCLUSIONS

3 mm MSIs placed in humans by a novice operator are likely to fail approximately 1/3 of the time. 

With the placement of more MSIs over time, failure rates decrease to approximately 20%.

Failures of 3 mm MSIs occur mostly between 2 and 4 weeks after insertion.

3 mm MSIs have acceptable levels of insertion torque, but low removal torque after eight weeks of healing.

3 mm MSIs tip during the first three weeks after insertion, with greater movements of the MSIs that fail.

Pain and discomfort experienced after 3 mm MSI placement is minimal and temporary.
